# Isopods infesting Atlantic bonefish (*Albula vulpes*) host novel viruses, including reoviruses related to global pathogens, and opportunistically feed on humans

**DOI:** 10.1017/S003118202400146X

**Published:** 2024-10

**Authors:** Tony L. Goldberg, Addiel U. Perez, Lewis J. Campbell

**Affiliations:** 1 Department of Pathobiological Sciences, School of Veterinary Medicine, University of Wisconsin-Madison, Madison, WI, USA; 2 Bonefish & Tarpon Trust, Miami, FL, USA

**Keywords:** Aegidae, *Aquareovirus*, Cymothooidea, Isopoda, recreational fishing, *Reovirales*, *Rocinela*, vector-borne disease, viruses, zoonoses

## Abstract

Isopods infest fish worldwide, but their role as disease vectors remains poorly understood. Here, we describe infestation of Atlantic bonefish (*Albula vulpes*) in Belize with isopods in two of three locations studied, with infestation rates of 15 and 44%. Isopods fed aggressively, and infested fish showed missing scales and scars. Gross morphologic and molecular phylogenetic analyses revealed the isopods to cluster within the family Aegidae and to be most closely related to members of the genus *Rocinela*, which are globally distributed micro-predators of fish. Metagenomic analysis of 10 isopods identified 11 viruses, including two novel reoviruses (*Reovirales*) in the families *Sedoreoviridae* and *Spinareoviridae*. The novel sedoreovirus clustered phylogenetically within an invertebrate-specific clade of viruses related to the genus *Orbivirus*, which contains arboviruses of global concern for mammal health. The novel spinareovirus clustered within the fish-infecting genus *Aquareovirus*, which contains viruses of global concern for fish health. Metagenomic analyses revealed no evidence of infection of bonefish with the novel aquareovirus, suggesting that viremia in bonefish is absent, low, or transient, or that isopods may have acquired the virus from other fish. During field collections, isopods aggressively bit humans, and blood meal analysis confirmed that isopods had fed on bonefish, other fish, and humans. Vector-borne transmission may be an underappreciated mechanism for aquareovirus transmission and for virus host switching between fish and other species, which has been inferred across viral families from studies of deep virus evolution.

## Graphical abstract

**Figure:**
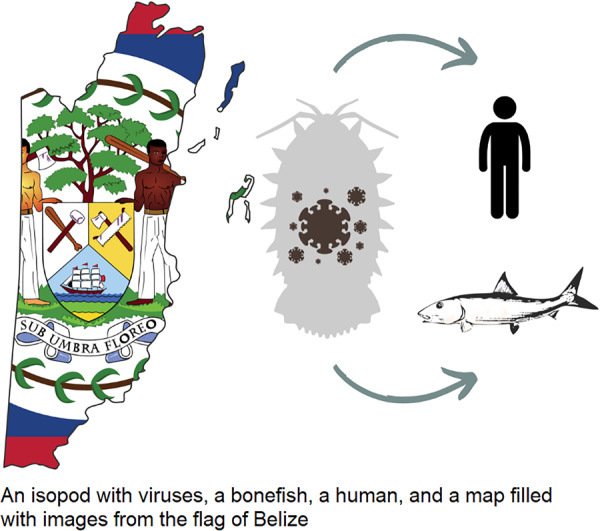

## Introduction

Parasitic and micro-predatory crustaceans are taxonomically diverse and globally distributed among a broad range of fish hosts (Smit *et al*., 2019*b*). These crustaceans include the taxa Amphipoda, Ascothoracida, Branchiura, Cirripedia, Copepoda, Isopoda, Ostracoda, Pentastomida and Tantulocarida, all of which have evolved intricate relationships with their hosts over long expanses of time (Klompmaker and Boxshall, 2015; Smit *et al*., 2019*a*). Such crustaceans are known to vector fish pathogens (Overstreet *et al*., 2009). However, much knowledge about crustaceans as disease vectors comes from studies of infestations/infections that threaten aquaculture. For example, a great deal is known about the transmission of salmonid diseases by copepods (‘sea lice’) because of economic damage suffered by aquaculture operations from these parasites and the pathogens they transmit (Overstreet *et al*., 2009; Hadfield and Smit, 2019; Bass *et al*., 2021). Far less is known about the natural history of other crustaceans and the pathogens they transmit within and among wild fish.

Fish also host myriad viruses, and the remarkable diversity fish viruses has only recently come to light (Harvey and Holmes, 2022). These viruses include the aetiological agents of fish diseases that threaten wild fisheries and aquaculture worldwide (Crane and Hyatt, 2011; Mondal *et al*., 2022). They also include distant relatives of notorious pathogens of humans and other terrestrial animals that threaten global health, such as ebolaviruses, coronaviruses, and influenza viruses (Parry *et al*., 2020; Geoghegan *et al*., 2021; Hierweger *et al*., 2021; Miller *et al*., 2021; Harvey and Holmes, 2022). Studies of the deep evolution of fish viruses and their relatives show frequent host switching among fish lineages and between fish and other vertebrate classes (Geoghegan *et al*., 2021; Harvey and Holmes, 2022). However, ecological mechanisms underlying such host switching are poorly understood, as are modes of transmission of most fish viruses in their natural hosts.

Herein is described an investigation of isopods infesting wild Atlantic bonefish (*Albula vulpes*) in Belize. Bonefish (*Albula* spp.) are a taxonomic complex of nearshore marine fish with a conserved morphology and physiology adapted for burst-speed swimming (Colborn *et al*., 1997; Murchie *et al*., 2011; Pickett *et al*., 2020). Bonefish are not propagated in captivity, but they sustain tourism economies around the world because of their value as targets of catch-and-release recreational angling (Adams, 2017; Smith *et al*., 2023). Discovered during an investigation of bonefish microbes and health across the Caribbean (Campbell *et al*., 2023*a*, 2023*b*; Castillo *et al*., 2024), the isopods were encountered at two localized study areas, and not at other nearby locations. Analyses of the isopods expand the known diversity of viruses that isopods carry and provide new information about the role of isopods as vectors for viruses of global concern to fish health. The feeding behaviour of these isopods, assessed by direct observation and blood meal analysis, reveals a potential role of isopods as facilitating viral host switching between fish and other vertebrates, including humans.

## Materials and methods

### Field studies

**Figure 1.**
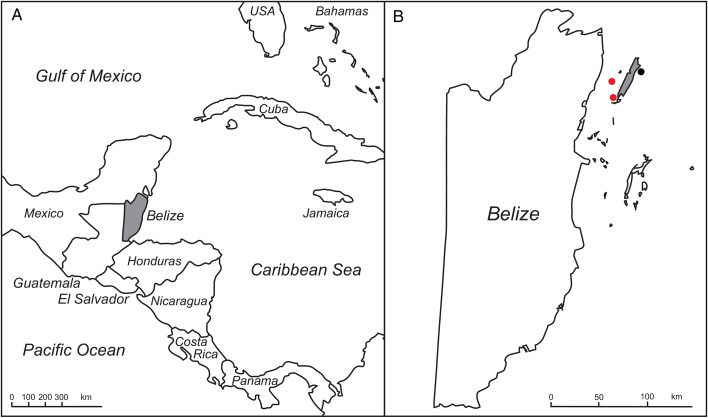
Map of sampling locations. Belize in Central America (A) and Ambergris Caye within Belize (B) are shaded grey. Circles in panel B indicate locations where bonefish were sampled. Red circles indicate locations with parasitic isopods of bonefish.

Bonefish were sampled as part of a pan-Caribbean study of bonefish microbes and health (Goldberg, 2019; Campbell *et al*., 2023*a*, 2023*b*; Castillo *et al*., 2024). Bonefish in Belize were captured from 28 June to 1 July 2019 at three sites: one site along the west coast (bay side) of Ambergris Caye, an island approximately 50 km northeast of mainland Belize, another site on the east coast (ocean side) of Ambergris Caye, and a third site at Blackadore Caye, approximately 8 km west of Ambergris Caye (Fig. 1). Fish were sampled and released as previously described (Campbell *et al*., 2023*b*). Briefly, fish were captured using seine nets, and blood samples of ⩽1% fish mass were obtained by caudal venipuncture and were processed and preserved in the field for molecular diagnostics, as previously described (Campbell *et al*., 2023*b*). All fish were examined by a veterinarian at the time of capture, and the presence of physical abnormalities such as ectoparasites or scars was recorded and photographed. Isopods were collected from fish using sterile forceps and placed into 1.2 mL sterile cryogenic vials containing 0.75 mL RNAlater nucleic acid preservation solution [Thermo Fisher Scientific, Waltham MA, USA]. Isopod samples were kept on ice in the field, frozen at −20°C within 6 h of collection and shipped to the USA for storage at −20°C until further analysis.

### Characterization of isopods

Isopods were photographed using a Leica Z16 APO dissecting microscope with apochromatic zoom objective and motor focus drive, using a Syncroscopy Auto-Montage System and software, as previously described (Young *et al*., 2016; Friant *et al*., 2022). Whole isopods were then individually surface-sterilized using dilute bleach (Binetruy *et al*., 2019) and homogenized in 0.5 mL Hanks' balanced salt solution in PowerBead tubes containing 2.38 mm metal beads [Qiagen, Hilden, Germany] using four 20 s cycles of bead-beating in an orbital homogenizer [Minibeadbeater, BioSpec Products, Bartlesville OK, USA]. DNA was extracted from 50 μL of isopod homogenate, and a portion of the cytochrome oxidase subunit 1 (cox1) gene was amplified using polymerase chain reaction (PCR) with barcoding primers LCO1490 (5′-GGTCAACAAATCATAAAGATATTGG-3′) and HC02198 (5′-TAAATCTCAGGGTGACCAAAAAATCA-3′) (Folmer *et al*., 1994) and sequenced as previously described (Ramírez-Martínez *et al*., 2021). Resulting nucleotide sequences were hand-aligned (no indels were present) with closely related, non-identical isopod sequences identified in GenBank. A phylogenetic tree was inferred using PhyML 3.056 (Guindon *et al*., 2010) with smart model selection (Lefort *et al*., 2017) and 1000 bootstrap replicates to assess statistical confidence in clades, and the resulting phylogenetic tree was displayed using FigTree v1.4.4.

### Isopod blood meal analysis

To identify hosts on which isopods had recently fed, metagenomic methods were used as described for other hematophagous arthropods (Brinkmann *et al*., 2016; Dumonteil *et al*., 2024; Mirza *et al*., 2024). Briefly, trimmed metagenomic data (see below) were subjected to *de novo* assembly as described below but prior to *in silico* subtraction of vertebrate sequences, then resulting contiguous sequences (contigs) were queried against vertebrate mitochondrial DNA sequences in GenBank using blastn (Altschul *et al*., 1990). Raw sequence reads from each isopod were then mapped to vertebrate mitochondrial DNA sequences thus identified using CLC Genomics Workbench version 23.0.2 [Qiagen, Hilden, Germany] to derive statistics on sequence coverage and percent nucleotide identity.

### Characterization of viruses

For virus identification, 250 *μ*L of isopod homogenate (see above) was subjected to virus enrichment by centrifugation and nuclease digestion to reduce non-encapsidated genetic material, as previously described for other hematophagous arthropods (Bennett *et al*., 2020; Ramírez-Martínez *et al*., 2021; Kamani *et al*., 2022). Total nucleic acids were extracted and converted to cDNA, and libraries were prepared using the Nextera XT DNA sample preparation kit [Illumina, San Diego, CA, USA] and sequenced on a MiSeq instrument [V3 chemistry, 600 cycle kit; Illumina, San Diego, CA, USA], also as previously described (Bennett *et al*., 2020; Ramírez-Martínez *et al*., 2021; Kamani *et al*., 2022). Resulting sequence reads were trimmed to a quality (Phred) score of ⩾Q30 and length ⩾50 using CLC Genomics Workbench, and reads were subtracted *in silico* of known contaminants, ribosomal sequences, the assembled *Ligia exotica* reference genome (GenBank GCA_002091915.1, to reduce isopod-derived sequences) and the assembled *Cyprinus carpio* genome (GenBank GCF_018340385.1, to reduce fish-derived sequences).

Remaining trimmed, decontaminated and ‘de-hosted’ sequences were subjected to *de novo* assembly using metaSPAdes v.3.15.5 (Nurk *et al*., 2017), and resulting contigs longer than 500 nt were queried using 6-frame translation against the NCBI non-redundant (nr) protein database using DIAMOND (Buchfink *et al*., 2015). Putative viral hits were then queried individually using blastn and blastp (Altschul *et al*., 1997) to the full GenBank nucleotide and protein databases, respectively, and ORF finder (Rombel *et al*., 2002) was used to verify deduced open reading frames. Contigs with homology to known viruses, an arrangement of uninterrupted open reading frames consistent with that of the putative viral family, and lack of non-viral sequences within the contig were carried forward. Bacteriophage sequences were excluded from further analysis, as the goal was to examine viruses capable of infecting eukaryotes. To infer the phylogenetic positions of reoviruses (see below), RNA-dependent RNA polymerase (RdRp) and outer capsid protein deduced amino acid sequences were aligned with homologous sequences in GenBank using MUSCLE (Edgar, 2004) implemented from EMBL-EMI (Madeira *et al*., 2022), then trimAl (Capella-Gutierrez *et al*., 2009) implemented from NGPhylogeny.fr (Lemoine *et al*., 2019) was applied to remove poorly aligned regions, and phylogenetic trees were inferred as described above.

## Results

### Field studies

**Figure 2.**
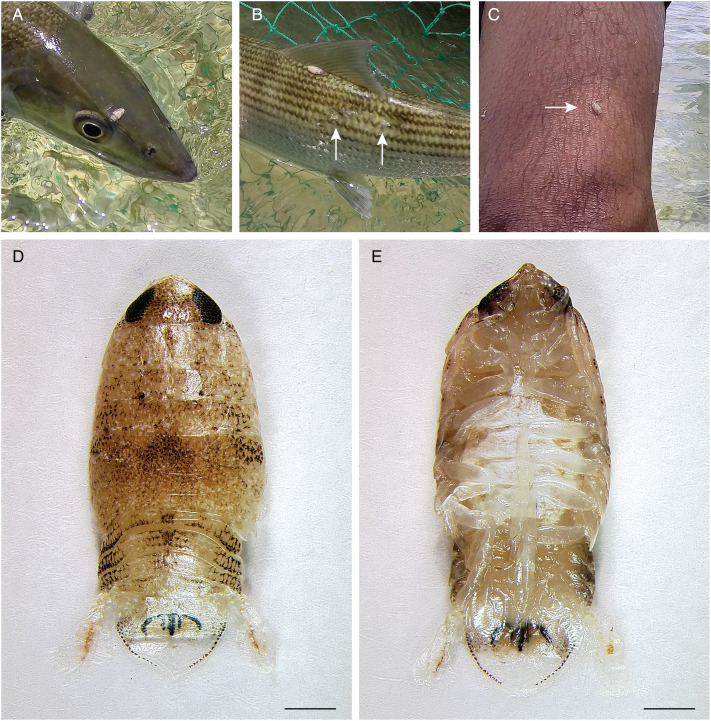
Isopods encountered in Belize. Isopod blood-feeding on a bonefish above its eye (A). Isopod blood-feeding on a bonefish below its dorsal fin, with scars and missing scales (arrows) indicating sites of prior infestation (B). Isopod (arrow) blood-feeding on the leg of a human wading in water during fish capture and processing (C). Dorsal (D) and ventral (E) montaged images of an isopod collected from a bonefish (scale bars = 1 mm).

A total of 91 bonefish were sampled on the west and east coasts of Ambergris Caye and near Blackadore Caye approximately 8 km east of Ambergris Caye (Fig. 1). Isopods were observed infesting 5/34 (14.71%) and 14/32 (43.75%) of bonefish at the site near Blackadore Caye and at the site on the west coast of Ambergris Caye, respectively (Fig. 1, Fig. 2A/B). Approximately 70% of isopods observed were visibly engorged. Fish at these locations had missing scales and scars indicative of prior isopod attachment and feeding (Fig. 2B). During sampling, isopods aggressively attached to people wading in the shallow water and successfully fed on these people (Fig. 2C). By contrast, no isopods or missing scales/scars (0/25; 0%) were observed on fish sampled at the location on the east side of Ambergris Caye (Fig. 1). Similarly, no isopods or missing scales/scars had been observed at other locations during a related study in which *A. vulpes* were sampled across nearly 2000 km of their Caribbean range (Campbell *et al*., 2023*a*, 2023*b*). Ten isopod specimens were collected for subsequent analysis.

### Characterization of isopods

**Figure 3.**
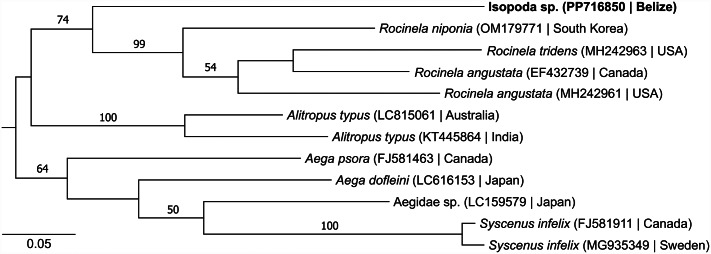
Maximum likelihood phylogenetic tree of isopods in the family Aegidae (Cymothooidea). The tree is based on a 602-position nucleotide sequence alignment of the mitochondrial cytochrome oxidase subunit 1 gene and a GTR + G + I model of molecular evolution. Taxon names are followed (in parentheses) by sequence accession number and country of origin. The isopod taxon identified in this study is highlighted in bold. The tree is midpoint rooted. Numbers beside branches indicate bootstrap values (percent) based on 1000 replicates; only values ⩾50% are shown. Scale bar indicates nucleotide substitutions per site.

Discussions with residents of Ambergris Caye revealed infestation of bonefish and humans with isopods to be a commonly known local phenomenon. Auto-Montage images of the isopods showed the typical size, streamlined body shape, and hooked pereopods of the free-living, smaller juvenile stage of fish-infesting isopods (Nagler and Haug, 2016; Williams and Bunkley-Williams, 2019), but finer characterization of morphological features (e.g. mouthparts) was unfortunately precluded by physical deformation resulting from freezing and storage in RNAlater buffer (Fig. 2D/E). Nevertheless, gross morphological features (e.g. the pigmentation pattern on the anterior of the pleotelson) suggest that the isopods may be members of the genus *Rocinela* (Aegidae) (Brusca and France, 1992) and possibly *R. signata*, which is widely distributed in the Western Atlantic (Bunkley-Williams *et al*., 2006; Silva *et al*., 2019; Aguilar-Perera and Nóh-Quiñones, 2022) and which attacks humans (Garzón-Ferreira, 1990). DNA sequences of cox1 were identical among all 10 specimens. Phylogenetic analysis revealed the newly generated isopod sequence to form a clade with previously sequenced members of the genus *Rocinela*, all of which were collected from locales in the Pacific Basin, but to be a divergent sequence within that clade (Fig. 3).

### Isopod blood meal analysis

Contiguous sequences from vertebrate mitochondrial genomes were recovered from seven of ten isopods, representing the seven visibly engorged isopods. Sequences from five of these isopods matched bonefish, sequences from one isopod were closest to the mangrove rivulus (*Kryptolebias marmoratus*), a fish common to nearshore habitats in the region (Taylor, 2012), and sequences from one isopod matched human (Table S2).

### Characterization of viruses

After quality and length trimming, a total of 19 143 401 sequences (average 1 914 340 sequences per isopod) of average length 127 were obtained. From these, 13 contigs were assembled, representing 11 viruses with varied genome composition in seven families (Table 1). When published sequences (81 815 890 reads) from 103 bonefish across the Caribbean were mapped at 90% stringency to these contigs (including sequences from bonefish from which the isopods described in this study were collected; Campbell *et al*., 2023*b*), no reads mapped. Viruses were named with sequential numbers following the unifying identifier ‘xkarip’ (pronounced ISH-ka-reep), which is the local name of the isoopds and the Mayan word for ‘fish flea.’

**Table 1.** Viruses in isopods parasitizing Atlantic bonefish

Virus	Abbrev	Contig length (nt)	Coverage^a^	Accession^b^	Closest match (host, location, year, accession)^c^	Genome^c^	Family^c^	Genus^c^	*E* value^c^	% ID (aa)^c^	Prevalence (%)^d^
Xkarip virus 1	XKRV-1	3859	23.5	PP816302	Grass carp reovirus (Grass carp, China, 2011, AJQ21748)	dsRNA (segmented)	*Spinareoviridae*	*Aquareovirus*	0E + 00	59.21	10
Xkarip virus 2	XKRV-2	529	1334.1	PP816313	Brine shrimp orbivirus 2 (Shrimp, China, u, UNI73877)	dsRNA (segmented)	*Sedoreoviridae*	unclassified	5E-40	26.21	80
Xkarip virus 2	XKRV-2	3651	2116.0	PP816314	Wufeng shrew orbivirus 1 (Shrew, China, u, WPV63685)	dsRNA (segmented)	*Sedoreoviridae*	unclassified	0E + 00	41.00	70
Xkarip virus 3	XKRV-3	2807	2.8	PP816315	Sarcosphaera coronaria partitivirus (Fungus, Turkey, 2019, QLC36816)	dsRNA (segmented)	*Partitiviridae*	unclassified	3E-24	42.86	10
Xkarip virus 4	XKRV-4	5888	1481.1	PP816316	Stinn virus (Mosquito, USA, 2017, QRW41701)	dsRNA (linear)	*Totiviridae*	unclassified	8E-33	26.30	60
Xkarip virus 5	XKRV-5	7107	922.8	PP816317	Wenzhou crab virus 2 (Crab, China, 2013, YP_010839346)	−ssRNA	*Chuviridae*	*Chuvivirus*	2E-36	30.52	10
Xkarip virus 6	XKRV-6	1678	260.7	PP816318	Hubei chuvirus-like virus 3 (Dragonflies/damselflies, China, 2013, YP_009337089)	−ssRNA	*Chuviridae*	*Scarabeuvirus*	0E + 00	31.02	20
Xkarip virus 7	XKRV-7	865	9.4	PP816319	Wufeng shrew chuvirus 1 (Shrew, China, u, OQ715564)	−ssRNA	*Chuviridae*	unclassified	6E-18	29.77	100
Xkarip virus 8	XKRV-8	989	56.3	PP816320	Canary chaphamaparvovirus 1 (Canary, Australia, 2023, WOP79074)	ssDNA (linear)	*Parvoviridae*	*Chaphamaparvovirus*	1E-11	35.20	10
Xkarip virus 8	XKRV-8	1799	11.5	PP816321	Tasmanian devil-associated chapparvovirus 1 (Tasmanian devil, Australia, 2017, YP_010798220)	ssDNA (linear)	*Parvoviridae*	*Chaphamaparvovirus*	5E-84	42.90	10
Xkarip virus 9	XKRV-9	4529	11.6	PP816322	Parvovirus myotis4232 (Bat, u, u, DBA48171)	ssDNA (linear)	*Parvoviridae*	unclassified	4E-59	34.59	100
Xkarip virus 10	XKRV-10	566	1.7	PP816323	Petrochirus diogenes giant hermit crab associated circular virus (Hermit crab, u, u, YP_009163897)	ssDNA (circular)	*Circoviridae*	unclassified	6E-40	48.91	20
Xkarip virus 11	XKRV-11	805	1.5	PP816324	Halhan virus 3 (Abalone, South Korea, 2017, YP_009552716)	unknown	unclassified	unclassified	2E-83	66.49	10

a Sequence coverage averaged across all isopods positive for a virus

b GenBank accession number of viral sequence from this study. Accession number for all 11 XKRV-1 segments are PP816302–PP816312.

c Closest match (u, unspecified), genome composition, family, genus, *E* value, and percent identity (amino acid, to the closest match) identified by querying the deduced amino acid sequence of the longest open reading frame on each contig against the NCBI nonredundant (nr) protein database using blastp

d Percent of isopods (*n* = 10) in which reads mapping to a virus sequence were detected

**Figure 4.**
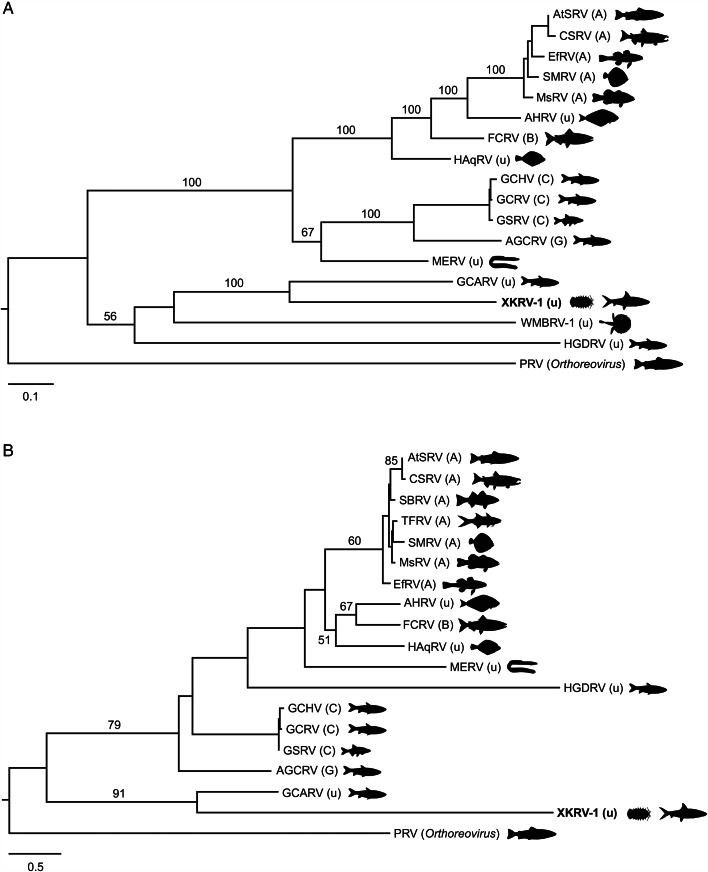
Maximum likelihood phylogenetic trees of aquareoviruses. Trees are based on amino acid alignments of 1271 positions and a LG + G + I model of molecular evolution for RNA-dependent RNA polymerase (A) and 228 positions and a Q.pfam + G + I model of molecular evolution for outer capsid protein (B). Letters in parentheses following virus abbreviations indicate the species designation of each virus (aquareovirus A, B, C, G or unclassified). Silhouettes represent host species for each virus. XKRV-1, the virus identified in this study, is highlighted in bold text. Trees are outgroup-rooted using piscine reovirus in the genus *Orthoreovirus.* Numbers beside branches indicate bootstrap values (percent) based on 1000 replicates; only values >50% are shown. Scale bars indicate amino acid substitutions per site. Full details of viruses are given in Table S1.

**Figure 5.**
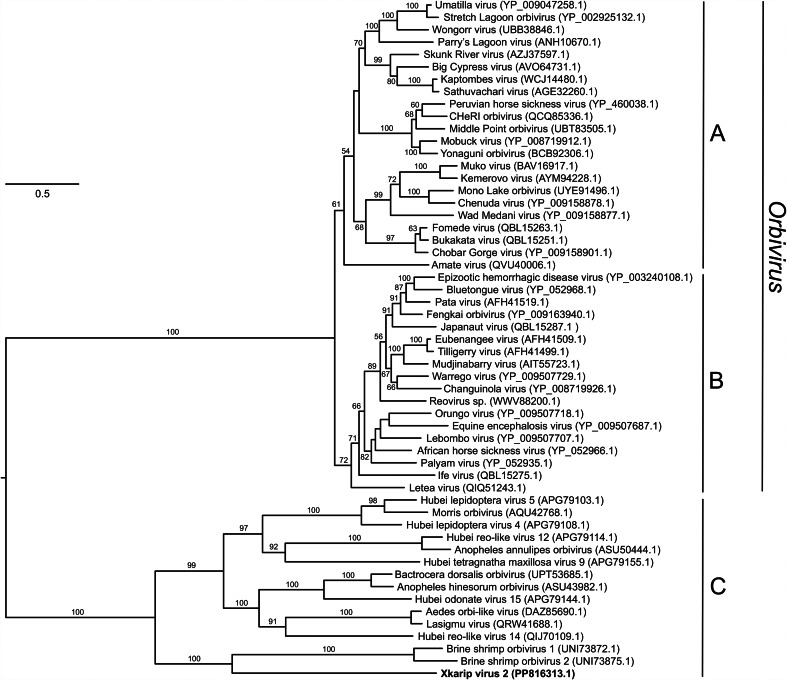
Maximum likelihood phylogenetic tree of reoviruses. The tree is based on a 1,091-position amino acid alignment of the RNA-dependent RNA polymerase and a LG + R + F model of molecular evolution. Taxon names are followed (in parentheses) by sequence accession number. Clades correspond to mosquito-borne and tick-borne viruses (A) and midge-borne viruses (B) within the genus *Orbivirus,* and a sister clade of currently unclassified invertebrate-specific reoviruses (C). The virus identified in this study, xkarip virus 2, is highlighted in bold. The tree is midpoint rooted. Numbers beside branches indicate bootstrap values (percent) based on 1000 replicates; only values >50% are shown. Scale bar indicates amino acid substitutions per site.

Of the 11 viruses identified, 10 were most closely related to viruses of arthropods, viruses from the feces of insectivorous or omnivorous birds and mammals, or viruses of fungi (Table 1). However, one virus, xkarip virus 1 (XKRV-1), was most closely related to a reovirus (*Reovirales*) in the family *Spinareoviridae*, genus *Aquareovirus*, which are viruses of fish (Fang, 2021). Analysis of the single isopod in which XKRV-1 was identified (XKRSP13) revealed a coding-complete 11-segment genome with between 10.3 and 51.8-fold sequence coverage (Table 2). The proteins encoded by each of the 11 viral segments were homologous to proteins of the exemplar virus Aquareovirus C, although at percent amino acid identities ranging from only 46.7 to 84.3% (Table 2). Phylogenetic analyses of complete XKRV-1 RNA-dependent RNA polymerase and outer capsid protein sequences showed the virus to be sister taxon to grass carp aquareovirus within a clade of currently unclassified (to species) aquareoviruses containing pathogens of global importance for fish health (Fig. 4, Table S1). Another reovirus, xkarip virus 2 (XKRV-2), was also identified, and this virus was most closely related to a virus in the family *Sedoreoviridae* (Table 1). Phylogenetic analysis of the complete XKRV-2 RNA-dependent RNA polymerase protein sequence showed it to be part of a clade of currently unclassified sedoreoviruses that is sister to viruses within the genus *Orbirus*, which contains mosquito/tick-borne and midge-borne viruses of global importance for mammal health (Fig. 5).

**Table 2.** Genomic characteristics of XKRV-1, a novel aquareovirus from isopod parasites of bonefish

Segment	Accession	Coverage^a^	Contig length (nt)^b^	Protein length (aa)^c^	Protein^d^	Product^d^	% id (AA)^d^	Accession (ref)^d^
1	PP816302	27.09	3919	1292	VP1	guanylyl/methyl transferase	66.42	NP_938060.1
2	PP816303	24.23	3859	1272	VP2	RNA-dependent RNA polymerase	84.25	NP_938061.1
3	PP816304	46.19	3712	1223	VP3	NTPase/helicase	71.01	NP_938062.1
4	PP816305	26.29	2215	725	VP5	core protein NTPase	63.27	NP_938064.1
5	PP816306	51.81	2163	711	NS1	non-structural protein NS1	60.38	NP_938063.1
6	PP816307	36.05	1999	652	VP4	outer capsid protein	67.47	NP_938065.1
7	PP816308	10.25	1507	488	VP56	fiber protein	55.88	ADJ75340.1
8	PP816309	43.31	1294	417	VP6	core protein	63.64	ADJ75342.1
9	PP816310	25.53	1090	349	VP38	sigma NS protein	77.78	ADJ75343.1
10	PP816311	44.23	1033	330	VP41	hypothetical protein	46.67	ADJ75341.1
11	PP816312	22.07	976	311	VP35	hypothetical protein	57.14	ADJ75344.1

a Average sequence coverage of the contiguous sequence (contig).

b Length of the full contiguous sequence of each viral segment (nuclootides).

c Length of the viral protein encoded by each segment (amino acids).

d Protein, protein product, and percent amino acid identity to Aquareovirus C, with accession numbers of each reference protein.

## Discussion

Investigation of bonefish from Belize revealed frequent infestation at two of three study sites with marine isopods, which also fed aggressively on humans. Eleven novel viruses were identified in the isopods, including two reoviruses, one of which is in the genus *Aquareovirus*, members of which threaten fish health worldwide. Blood meal analysis of isopods confirmed that they had fed on bonefish, other fish, and humans. These findings expand our knowledge of the diversity of viruses carried by parasitic and micro-predatory isopods. These findings also suggest that vector-borne transmission by isopods may be an underappreciated mechanism for the maintenance of viruses in fish and for the transmission of viruses between fish and other classes of vertebrates.

Biting arthropods are globally important for their role in transmitting and spreading vector-borne diseases, including some of the most consequential human and animal pathogens (Athni *et al*., 2021; Cuthbert *et al*., 2023; de Souza and Weaver, 2024). Historically, most research on such arthropods has focused on biting insects (e.g. mosquitoes) and arachnids (e.g. ticks) in terrestrial ecosystems because of the importance of the diseases these vectors transmit for human and domestic animal health (Swei *et al*., 2020; Cuthbert *et al*., 2023). However, there exists in nature a multitude of ‘neglected vectors’ that are equally capable of biologically vectoring pathogens, including viruses, although they remain far less studied (Baldacchino *et al*., 2014; Sick *et al*., 2019; Weitzel *et al*., 2020). Such neglected vectors may be particularly important in aquatic and marine animal ecosystems, where the disease-transmitting life stages of ticks and mosquitoes do not occur, except in unusual circumstances (Miyake *et al*., 2019).

Isopods infest fish globally and are among the most numerous of the parasitic crustacean taxa, and new isopods are discovered and characterized frequently (Smit *et al*., 2014; Boxshall and Hayes, 2019). Morphologically, the isopods from Belize are consistent with members of the genus *Rocinela*, which are widely distributed globally (Brusca and France, 1992), and may be *R. signata*, which infests diverse fishes from the south-eastern USA to southern Brazil (Bunkley-Williams *et al*., 2006; Silva *et al*., 2019; Aguilar-Perera and Nóh-Quiñones, 2022). Phylogenetically, the isopods clustered within the Aegidae as sister taxon to a clade containing members of the genus *Rocinela*. This phylogenetic position is consistent with the isopods being *R. signata*, especially since the other three previously sequenced *Rocinela* species originated from Pacific Basin locales, whereas Belize is in the Atlantic Basin. The behaviour of the isopods is also consistent with this taxonomy, based on a previous report from Colombia describing *R. signata*'s attachment to humans as ‘tenacious’ and leading to successful blood-feeding (Garzón-Ferreira, 1990). Unfortunately, the isopods in Belize were encountered unexpectedly and were preserved using available materials, which precluded fine-scale morphological description. Should a vouchered specimen of *R. signata* be sequenced in the future, it might confirm the identity of the isopods in this study.

Of the 11 viruses identified, 10 were associated with invertebrates, including crustaceans, other marine invertebrates, and invertebrates in the diets of vertebrates. This finding is consistent with previous studies showing isopod viromes to be dominated by invertebrate-specific viruses (Kuris *et al*., 1979; Johnson, 1984; Overstreet *et al*., 2009; Piégu *et al*., 2014; Bojko *et al*., 2020). The aquareovirus XKRV-1 was a clear exception to this pattern. All known members of the genus *Aquareovirus* infect fish, sometimes causing serious disease (Lupiani *et al*., 1995; Fang *et al*., 2021). XKRV-1 is most likely a fish-infecting virus, and its presence in the isopod likely indicates acquisition *via* feeding. There was no evidence of XKRV-1 in the blood of 103 bonefish from across the Caribbean, including the fish in Belize from which isopods were collected, nor of any other known bonefish viruses (Campbell *et al*., 2023*b*). This finding could indicate no, low, or transient XKRV-1 viremia in bonefish, or that the isopod acquired XKRV-1 from a previous fish host. However, *A. vulpes* was identified as the blood meal host of the isopod in which XKRV-1 was found. Therefore, if the isopod did acquire XKRV-1 from another species than bonefish, the virus must have persisted in the isopod through its acquisition of a subsequent bonefish blood meal. A hallmark of vectored viruses is persistence/replication in their vectors, sometimes even transstadially (Lequime and Lambrechts, 2014; Lange *et al*., 2024). Moreover, XKRV-1 was found at a low rate of infection (10%), which is typical of arboviruses in their vectors (Kirstein *et al*., 2021; Lange *et al*., 2024).

Another reovirus identified in the isopods, XKRV-2, provides an informative contrast to XKRV-1. XKRV-2 is part of a clade of reoviruses within the family *Sedoreoviridae* that is sister to vector-borne viruses in the genus *Orbivirus*, within which viruses form two clades based on whether they are mosquito-borne/tick-borne or transmitted by biting culicoid midges (Matthijnssens *et al*., 2022*b*). Viruses in this as-yet unclassified sister clade are not known to be transmitted to vertebrates and are thus likely invertebrate-specific. Reo-like viruses have been described in shellfish (bivalves, crabs and shrimp) but their classification remains unclear (Lupiani *et al*., 1995), and invertebrate-specific viruses appear (albeit from limited studies) to be common in isopods (Kuris *et al*., 1979; Johnson, 1984; Overstreet *et al*., 2009; Piégu *et al*., 2014; Bojko *et al*., 2020). XKRV-2 was also detected in 70–80% of isopods (in contrast to XKRV-1, which was detected in only one isopod), and high infection rates such as these are typical for non-vector-borne, arthropod-specific viruses, which are thought to be vertically transmitted (McLean *et al*., 2015; Calisher and Higgs, 2018; Carvalho and Long, 2021). In aggregate, these data show that isopods can carry vertebrate-infecting and arthropod-specific reoviruses simultaneously.

Despite decades of research on the molecular biology and replication of aquareoviruses since their first isolation in 1979, modes transmission of these viruses in nature remain surprisingly poorly understood (Lupiani *et al*., 1995; Samal, 2011; Fang *et al*., 2021). Aquareoviruses such as grass carp reovirus can be experimentally transmitted *via* immersion, and horizontal and vertical transmission have been inferred from epidemiological patterns observed during outbreaks (Zhang *et al*., 2021). Given the known vector-borne transmission mode of other reoviruses (Matthijnssens *et al*., 2022*a*, 2022*b*), it is plausible that aquareoviruses could undergo vector-borne transmission as well. Isopods serve as vectors of fish haemogregarines and other apicomplexan parasites (Hadfield and Smit, 2019; Sikkel *et al*., 2020). However, direct information on vectoring of viruses by isopods is scant. Transmission of virus-like particles in parasitic isopods to the crabs they parasitize has been hypothesized, but no direct evidence of such transmission exists (Kuris *et al*., 1979; Hadfield and Smit, 2019). Cymothoid isopods may vector lymphocystis disease virus (*Iridoviridae*), and gnathiid isopods may vector viral erythrocytic necrosis virus (*Iridoviridae*) (Hadfield and Smit, 2019). Nevertheless, isopods have adaptations for parasitism that viruses could exploit, such as salivary antihemostatic, anti-inflammatory, and immunomodulatory molecules (‘spit’) injected into hosts during feeding (Li *et al*., 2019), which enhances virus transmission in mosquitoes, ticks and sandflies (Conway *et al*., 2014; Schneider *et al*., 2021; Maqbool *et al*., 2022; Wang *et al*., 2024). Additional investigations of XKRV-1 and similar viruses in isopods might prove informative, such as tissue distribution studies to determine whether a virus is localized to the salivary glands or experimental feeding and transmission studies if viruses can be isolated.

The isopods in Belize fed aggressively and non-specifically, as evidenced by field observations and blood meal analysis indicating bonefish, another fish related to the mangrove rivulet, and a human as blood hosts. Parasitic crustaceans such as isopods might therefore serve as useful systems for pathogen biomonitoring (aka ‘xenosurveillance’ or ‘xenomonitoring’) in marine ecosystems, as has been proposed for hematophagouos arthropods in terrestrial ecosystems (Cameron and Ramesh, 2021; Rowan *et al*., 2023; Valente *et al*., 2023). They may also have direct effects on fish health and reproduction (Poore and Bruce, 2012), which could be particularly important in locations such as Belize where finfish are a food source and support economies based on recreational fishing. In this light, it is intriguing that the isopods were encountered only on the west coast of Ambergris Caye and at Blackadore Caye (also to the west of Ambergris Caye), and not off the east coast of Ambergris Caye. A previous study found surprisingly large differences in bacterial community composition on the gills of these same bonefish between the east (ocean side) and west (bay side) of Ambergris Caye (Campbell *et al*., 2023*a*). Isopods such as those described herein may favour certain habitat types or substrates (e.g. the locations where they were encountered during this study had deep layers of fine silt) and may thus be useful for local-scale biomonitoring. Parasites communities, which include isopods, have been proposed as indicators of habitat connectivity in coral reef ecosystems in schoolmasters (*Lutjanus apodus*) in study sites in Mexico, near those described here in Belize (Hernández-Olascoaga *et al*., 2022).

Fish host relatives of many viruses of global health importance to humans and other animals. For example, studies of fish have identified viruses in the families *Arenaviridae*, *Coronaviridae*, *Filoviridae*, *Flaviviridae*, *Hantaviridae, Hepeviridae*, *Matonaviridae*, *Orthomyxoviridae*, *Paramyxoviridae*, *Poxviridae*, and *Rhabdoviridae*, all of which contain pathogens responsible for human epidemics or pandemics (Geoghegan *et al*., 2018, 2021; Parry *et al*., 2020; Grimwood *et al*., 2021, 2023; Miller *et al*., 2021; Lensink *et al*., 2022; Perry *et al*., 2022; Xi *et al*., 2023; Ford *et al*., 2024). Analyses of deep virus evolution in these same studies consistently infer topological incongruity between host and viral phylogenies, implying frequent ancestral viral exchange among vertebrate host taxa. Despite this generalized pattern, the mechanisms by which fish exchange viruses with other vertebrates remain obscure. Vector-borne transmission is a plausible mechanism for such inter-class viral transmission. The propensity of some isopods to feed on humans aggressively, even when fish are present, also suggests a way that humans could be exposed to fish viruses. Contemporary zoonotic viruses of fish are unknown, except in the case of contamination of consumed fish with human gastrointestinal viruses (Ziarati *et al*., 2022). The findings presented herein demonstrate a mechanism whereby zoonotic transmission of virus naturally hosted by fish could conceivably occur.

## Supporting information

Goldberg et al. supplementary material 1 Goldberg et al. supplementary material

Goldberg et al. supplementary material 2 Goldberg et al. supplementary material

## Data Availability

The isopod cox1 sequence was deposited in the NIH National Center for Biotechnology Information (NCBI) GenBank database under accession number PP716850. All raw metagenomic sequence reads were deposited in the NCBI Sequence Read Achieve under BioProject PRJNA1103849. All assembled virus genome sequences were deposited in GenBank under accession numbers PP816302-PP816324.

## Supplementary material

The supplementary material for this article can be found at https://doi.org/10.1017/S003118202400146X.
